# Conversational Artificial Intelligence for Integrating Social Determinants, Genomics, and Clinical Data in Precision Medicine: Development and Implementation Study of the AI-HOPE-PM System

**DOI:** 10.2196/76553

**Published:** 2025-10-10

**Authors:** Ei-Wen Yang, Brigette Waldrup, Enrique Velazquez-Villarreal

**Affiliations:** 1 Polyagent, San Francisco, CA, United States; 2 Beckman Research Institute, Duarte, CA, United States; 3 City Of Hope National Medical Center, 1500 E Duarte Road, Duarte, CA, 91010, United States, 1 6262187162

**Keywords:** artificial intelligence, LLM, AI agent, bioinformatics, cancer, genomics, precision medicine, social determinants of health, large language model

## Abstract

**Background:**

Integrating clinical, genomic, and social determinants of health (SDOH) data is essential for advancing precision medicine and addressing cancer health disparities. However, existing bioinformatics tools often lack the flexibility to perform equity-driven analyses or require significant programming expertise.

**Objective:**

We developed AI-HOPE-PM (Artificial Intelligence Agent for High-Optimization and Precision Medicine in Population Metrics), a conversational artificial intelligence system designed to enable natural language–driven, multidimensional cancer analysis. This study describes the development, implementation, and application of AI-HOPE-PM to support hypothesis testing that integrates genomic, clinical, and SDOH data.

**Methods:**

AI-HOPE-PM leverages large language models and Python-based statistical scripts to convert user-defined natural language queries into executable workflows. It was evaluated using curated colorectal cancer datasets from The Cancer Genome Atlas and cBioPortal, enriched with harmonized SDOH variables. Accuracy of natural language interpretation, run time efficiency, and usability were benchmarked against cBioPortal and UCSC Xena.

**Results:**

AI-HOPE-PM successfully supported case-control stratification, survival modeling, and odds ratio analysis using natural language prompts. In colorectal cancer case studies, the system revealed significant disparities in progression-free survival and treatment access based on financial strain, health care access, food insecurity, and social support, demonstrating the importance of integrating SDOH in cancer research. Benchmark testing showed faster task execution compared to existing platforms, and the system achieved 92.5% accuracy in parsing biomedical queries.

**Conclusions:**

AI-HOPE-PM lowers technical barriers to integrative cancer research by enabling real-time, user-friendly exploration of clinical, genomic, and SDOH data. It expands on prior work by incorporating equity metrics into precision oncology workflows and offers a scalable tool for supporting disparities-focused translational research. Five videos are included as multimedia appendices to demonstrate platform functionality in real-world scenarios.

## Introduction

Health care is being transformed by comprehensive precision medicine, which personalizes treatment based on individual differences in genetics, environment, and lifestyle [1,2]. Alongside this shift, there is growing recognition of the critical role social determinants of health (SDOH) play in shaping disease outcomes and access to care [2-5]. To advance both scientific discovery and health equity, integrating clinical, genomic, and SDOH data is imperative for uncovering disease mechanisms, enhancing treatment effectiveness, and reducing disparities—especially among underserved populations. However, several challenges remain: data silos, the need for specialized expertise in multiomics analysis, and the underrepresentation of diverse populations in existing datasets all continue to hinder the equitable realization of precision medicine [6-9].

The complexity of cancer research workflows demands seamless integration of molecular profiles, clinical metadata, and population-level variables such as race, ethnicity, income, health literacy, and access to care. Although web-based tools like cBioPortal [10] and UALCAN [11] offer structured platforms for querying public cancer datasets such as The Cancer Genome Atlas (TCGA) [12], they operate within predefined analytical frameworks and require users to manually conduct multistep filtering, stratification, and statistical interpretation [13-16]. These limitations restrict the flexibility needed to explore hypothesis-driven, context-specific research questions—especially those involving SDOH variables critical for addressing health equity.

Meanwhile, emerging artificial intelligence (AI)–based tools like CellAgent [17] and AutoBA [18] have begun to explore the potential of large language models (LLMs) in bioinformatics workflows [19-22]. However, these systems often focus solely on genomic data and lack the capacity to simultaneously integrate clinical and SDOH variables, thereby limiting their utility in advancing equitable biomedical research.

**Figure 1.**
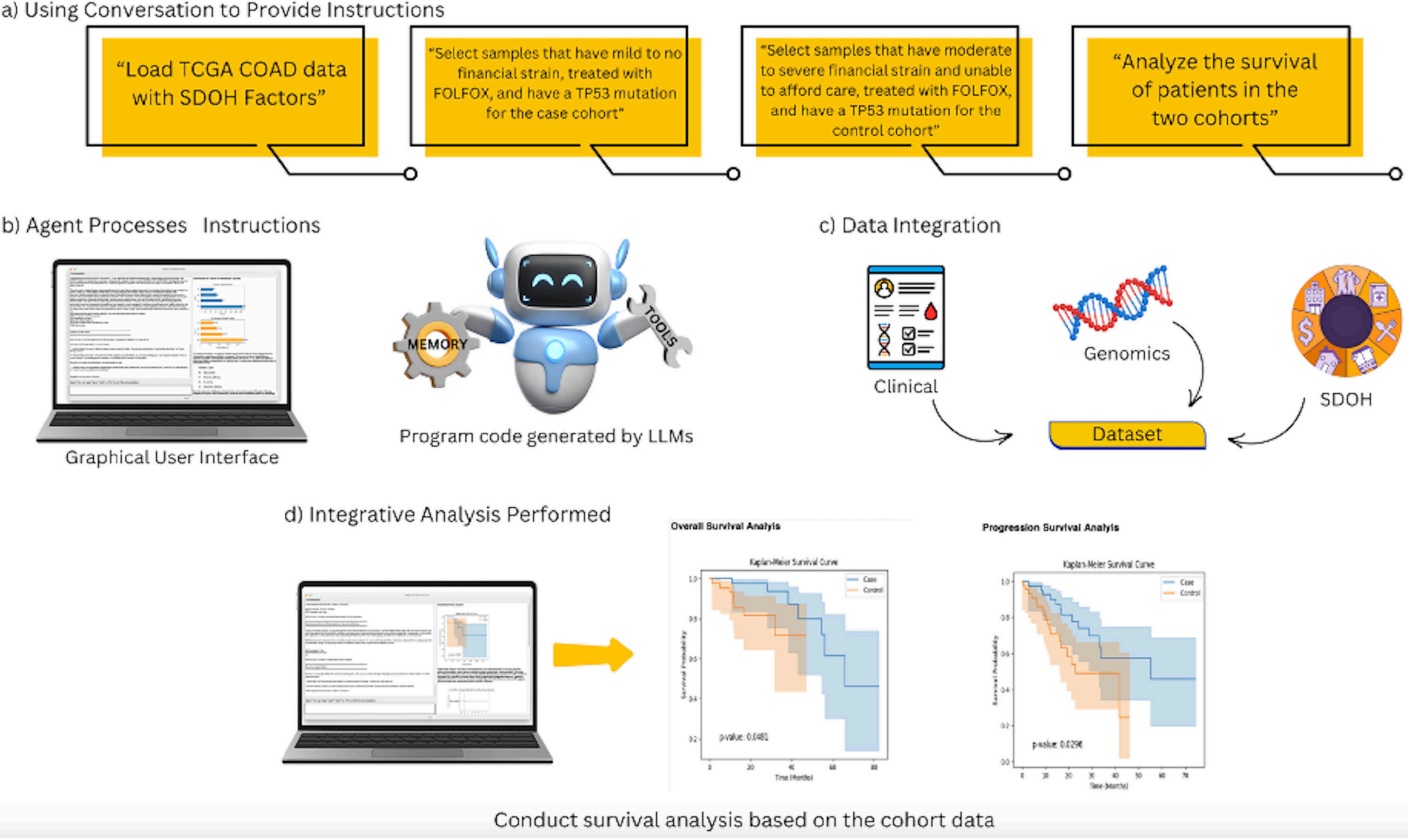
Overview of AI-HOPE-PM (Artificial Intelligence Agent for High-Optimization and Precision Medicine in Population Metrics) workflow. LLM: large language model; SDOH: social determinants of health; TCGA: The Cancer Genome Atlas.

Motivated by these gaps, we introduced AI-HOPE-PM (Artificial Intelligence Agent for High-Optimization and Precision Medicine in Population Metrics), a novel LLM-powered conversational agent designed to democratize access to integrative bioinformatics analysis. AI-HOPE-PM allows users—regardless of technical background—to conduct robust, multidimensional cancer research using natural language queries. As illustrated in Figure 1, the platform employs natural language processing, retrieval-augmented generation, and Python-based bioinformatics pipelines to translate user queries into reproducible and explainable analyses. This includes case–control comparisons, survival modeling, and stratified multiomics analysis—all without requiring code or manual data preprocessing.

Unlike traditional graphical user interface (GUI) tools, AI-HOPE-PM supports complex, user-defined queries such as “Analyze FOLFOX-treated colorectal cancer (CRC) patients with TP53 mutations and varying levels of financial strain.” The system autonomously identifies relevant data, filters patient cohorts, integrates clinical treatment and genomic mutation data with socioeconomic context, and generates statistical visualizations, survival curves, and interpretative summaries. By enabling real-time, dynamic exploration of clinical-genomic-SDOH interactions, AI-HOPE-PM simplifies complex workflows and enhances the translational relevance of precision oncology research. This work builds on our previously developed platform, AI-HOPE [23], a conversational AI agent designed to support natural language-driven integration of clinical and genomic data for precision medicine research. While AI-HOPE demonstrated effective local analysis of structured datasets and addressed key bioinformatics needs, it did not incorporate SDOH or population-level variables critical to health equity research. AI-HOPE-PM extends this foundation by integrating SDOH data and supporting population-aware case-control analyses, enabling researchers to interrogate disparities across both molecular and social axes. To evaluate its performance, AI-HOPE-PM is being benchmarked against established tools such as cBioPortal and UCSC Xena [24]. The benchmarking involves assessing run time efficiency, reproducibility, and usability. In contrast to tools that require step-by-step configuration, AI-HOPE-PM offers streamlined execution of advanced bioinformatics pipelines through LLM-guided user interaction, significantly lowering barriers to data exploration and hypothesis testing.

By bridging the gap between data complexity and user accessibility, AI-HOPE-PM offers a scalable, inclusive, and equitable AI framework for biomedical discovery. Its ability to integrate clinical, genomic, and SDOH variables addresses the long-standing need for tools that not only generate high-quality insights but also promote diversity and inclusiveness in biomedical research.

To address the limitations of current bioinformatics tools and advance equity in translational precision medicine, this study introduces AI-HOPE-PM—a novel conversational AI platform purpose-built to integrate clinical, genomic, and SDOH data through natural language interaction. The aim of this paper is to describe the development, implementation, and application of AI-HOPE-PM for multidimensional cancer analysis, with a focus on its ability to democratize data exploration, reduce technical barriers, and enable equity-driven hypothesis testing. Specifically, we demonstrate how AI-HOPE-PM enables real-time, case-control, and survival analyses that incorporate SDOH variables such as financial strain, food insecurity, health care access, and health literacy, alongside genomic and clinical features. By benchmarking its performance and illustrating its use through case studies in CRC, we highlight the platform’s potential to accelerate disparities-focused research, improve biomarker discovery, and support inclusive precision oncology.

## Methods

### Development of AI-HOPE-PM and Data Sources

AI-HOPE-PM is a conversational AI platform designed to advance translational precision oncology by enabling users to perform integrative bioinformatics analyses through plain-language queries. The system is built on a retrieval-augmented generation framework—a method that enhances response accuracy by retrieving relevant information from structured datasets—and a fine-tuned biomedical LLM (LLaMA 3). Behind the scenes, the platform uses Python-based scripts to carry out statistical analyses and genomic data processing.

To enable robust analyses, we used curated multimodal datasets from TCGA, AACR Project GENIE, and cBioPortal. These datasets included harmonized clinical, genomic, and demographic variables. In addition, we generated synthetic SDOH variables using a validated Python script, guided by a literature-informed framework. These SDOH features included health care access, financial strain, food insecurity, social support, and health literacy. All datasets were preprocessed into standardized tab-delimited formats with annotated metadata describing each variable type. A full list of variables analyzed—including over 200 clinical, genomic, treatment, and SDOH fields—is publicly available [25], which also contains the source code, example queries, simulated data, and documentation for reproducing all analyses.

### Workflow and Natural Language Interface

Users interact with AI-HOPE-PM via a GUI that accepts plaintext queries. The system interprets these queries using a natural language processing engine to define analytic tasks, including loading a dataset, stratifying cohorts based on genomic or SDOH features, and performing statistical analyses such as survival modeling or odds ratio testing. The resulting structured commands are executed programmatically, streamlining workflows that typically require multiple manual steps or coding expertise.

### Evaluation and Validation of System Accuracy

We evaluated AI-HOPE-PM’s query interpretation accuracy using 100 natural language prompts that reflected diverse real-world research scenarios in clinical genomics and health disparities. A team of expert reviewers established ground truth interpretations for each query to assess system performance. AI-HOPE-PM achieved an overall accuracy of 92.5%, with near-perfect accuracy (99.1%) for single-variable queries and strong performance (88.4%) for more complex, multivariable prompts. Most errors stemmed from ambiguous phrasing (eg, nonspecific end points), syntactic inconsistencies (eg, nested logic), or misalignment between user language and system variable mappings. To address these issues, AI-HOPE-PM integrates built-in clarification prompts and applies a domain-specific ontology to harmonize terminology and guide users toward more structured input. Future development will focus on improving the natural language understanding engine and refining internal mapping algorithms to further enhance accuracy and reproducibility.

To confirm the analytical fidelity of AI-HOPE-PM, we cross-validated its survival analyses, odds ratio outputs, and cohort stratifications against manually performed analyses previously published by our group using similar datasets and variables. These included studies investigating CRC disparities based on *TP53*, *APC*, and *KRAS* mutation status, treatment modality, and SDOH factors across TCGA and cBioPortal cohorts. The results generated by AI-HOPE-PM were consistent with those from traditional statistical pipelines in terms of hazard ratios, *P* values, and overall survival trends. This validation step supports the platform’s accuracy in replicating established findings and reinforces its reliability as a tool for real-time, natural language–driven bioinformatics analyses.

Although we benchmarked AI-HOPE-PM against established platforms such as cBioPortal and UCSC Xena, it is important to acknowledge that these platforms function through traditional GUIs requiring multistep, click-based interactions. This structural difference makes direct comparisons with AI-HOPE-PM—an intelligent, conversational AI system—challenging. In cBioPortal and Xena, executing multilayered queries or stratified analyses may involve multiple browser windows, dropdown menus, and manual dataset subsetting. In contrast, AI-HOPE-PM enables users to perform similar tasks via a single plain-language prompt, streamlining the process and reducing complexity. While speed remains an advantage for AI-HOPE-PM, we also validated its outputs through comparisons with previously published manual analyses, ensuring consistency and analytical fidelity. This intelligent design is intended to reduce the technical barrier for researchers and support scalable, real-time hypothesis generation in precision medicine Multimedia Appendix 1.

To ensure robustness in handling natural language variability, AI-HOPE-PM incorporates an interactive clarification mechanism that prompts users for additional input when queries are ambiguous or underspecified. Common edge cases include vague end points (eg, “better outcomes”), undefined comparison groups, or syntactic inconsistencies (eg, nested logic). In these instances, the system pauses execution and requests clarification through a structured prompt. Furthermore, AI-HOPE-PM uses a curated biomedical ontology to harmonize synonymous terms and align user inputs with internal variable definitions. These strategies support resilient query interpretation and maintain analytical fidelity across diverse and potentially ambiguous user queries (Multimedia Appendix 2).

### Benchmarking and Comparative Analysis

To assess usability and speed, we benchmarked AI-HOPE-PM against existing platforms including cBioPortal and UCSC Xena. Biomedical researchers were asked to complete tasks such as dataset loading, filtering based on genomic or SDOH attributes, and initiating analyses. Task durations were measured using stopwatch protocols. AI-HOPE-PM consistently outperformed traditional tools in terms of execution time and ease of use, owing to its automation and intuitive language-driven interface.

To evaluate the capacity of AI-HOPE-PM to integrate and analyze SDOH alongside clinical and genomic data, we developed a set of simulated SDOH variables. These variables—including financial strain, food insecurity, social support, health literacy, and insurance access—were generated using a Python-based simulation framework informed by published epidemiological distributions and associations relevant to cancer outcomes. The simulation approach was designed to mirror the variability and prevalence observed in real-world populations [26], thereby enabling realistic case–control stratifications and hypothesis testing. Although these SDOH variables are simulated, they serve as a pragmatic proxy in the absence of widely available, high-quality, individual-level SDOH data within public genomic datasets. For full transparency and reproducibility, the simulation scripts are publicly available [25]. Future validation studies using empirical SDOH data from institutional and community-linked datasets are planned to further refine and expand the platform’s capabilities.

### Statistical Analysis and Report Generation

The platform supports several statistical methods commonly used in cancer genomics, including Kaplan-Meier survival analysis with log-rank testing, Cox proportional hazards regression, and odds ratio calculations for categorical comparisons. Output includes plots such as survival curves and forest plots, accompanied by narrative summaries that describe the findings in context. All outputs are backed by reproducible Python code logs, which are stored internally and can be exported for validation or inclusion in publications [13-15].

### Usability Study and Accessibility

A formal usability study is underway to evaluate AI-HOPE-PM’s effectiveness and accessibility for biomedical researchers. Participants are comparing its interface, output quality, and query interpretation capabilities with those of GUI-based tools and other AI-driven platforms. While we did not perform head-to-head comparisons with generative systems such as CellAgent or AutoBA due to differing scopes, AI-HOPE-PM’s unique ability to integrate SDOH, clinical, and genomic data positions it as a novel tool for equitable and scalable precision medicine research.

To preliminarily assess usability, we conducted a small-scale case study involving six non-bioinformatician users, including oncology fellows and public health researchers. Participants were asked to perform common research tasks using AI-HOPE-PM—such as loading datasets, selecting cohorts by genomic and social variables, and running survival analyses—using only natural language queries. All users completed the tasks successfully, with positive feedback highlighting the intuitive interface, rapid execution, and elimination of the need for coding expertise. These findings provide initial validation of the platform’s accessibility to diverse research users.

### Ethical and Privacy Considerations

As with any LLM-based system, AI-HOPE-PM is susceptible to biases and potential hallucinations, particularly when interpreting complex or underspecified queries. To mitigate these risks, the system integrates domain-specific ontologies and harmonized variable dictionaries to reduce misinterpretation and support consistent query resolution. Additionally, the platform’s built-in clarification prompts serve as a real-time validation mechanism, prompting users to confirm or refine ambiguous instructions. While simulated SDOH-genomic interactions provide a useful testing framework, future efforts will emphasize empirical validation using real-world datasets to reduce confounding.

To address privacy concerns when working with sensitive real-world SDOH variables—such as insurance status, ethnicity, and income—AI-HOPE-PM is designed to operate as a secure, local AI system deployed within institutional infrastructures. Unlike cloud-based models that may transmit data externally, AI-HOPE-PM processes all data on-site, minimizing the risk of exposure or unauthorized access. This local deployment model supports compliance with data protection regulations, including the Health Insurance Portability and Accountability Act and the General Data Protection Regulation, where applicable. In future iterations, we plan to integrate customizable privacy modules and access controls to align with institutional review board protocols and ensure ethical handling of sensitive population-level health data.

To mitigate the risk of hallucinations and enhance the reliability of AI-HOPE-PM’s outputs, the platform incorporates several ethical safeguards. First, the system leverages domain-specific biomedical ontologies to align user queries with validated clinical and genomic concepts, reducing the likelihood of misinterpretation. Second, AI-HOPE-PM includes built-in prompts that clarify ambiguous user input, supporting more accurate query resolution. We also plan to implement human-in-the-loop verification workflows and bias detection modules, which will allow researchers to review, confirm, and flag generated outputs prior to downstream use. These strategies collectively enhance interpretability, accountability, and user trust in the AI-driven analytical process.

Given the sensitivity of SDOH data, especially variables such as insurance status, ethnicity, or socioeconomic conditions, AI-HOPE-PM is currently designed as a locally deployed system to prevent data exposure through external servers or third-party services. This architecture ensures that no identifiable information is shared beyond institutional firewalls. For future deployments that may involve real-world SDOH data, we plan to incorporate privacy-preserving methods including data deidentification, access controls, and secure computation protocols. All future iterations will comply with established data protection regulations such as the Health Insurance Portability and Accountability Act and the General Data Protection Regulation, ensuring responsible and ethical use of sensitive population-level data.

## Results

By converting natural language instructions into executable bioinformatics workflows, AI-HOPE-PM enabled seamless integration and analysis of clinical, genomic, and SDOH data within CRC datasets. The platform’s ability to interpret user queries and automate complex analyses demonstrated its effectiveness in supporting multidimensional, translational cancer research. Through its intuitive conversational interface, the system dynamically classified patient samples into case and control cohorts based on user-defined criteria. These criteria encompassed gene mutation status, treatment regimens, SDOH attributes, and demographic variables, facilitating highly customizable stratifications. The system autonomously performed statistical analyses—including prevalence estimation, odds ratio tests, and survival modeling—and generated comprehensive visualizations and interpretable reports.

**Figure 2.**
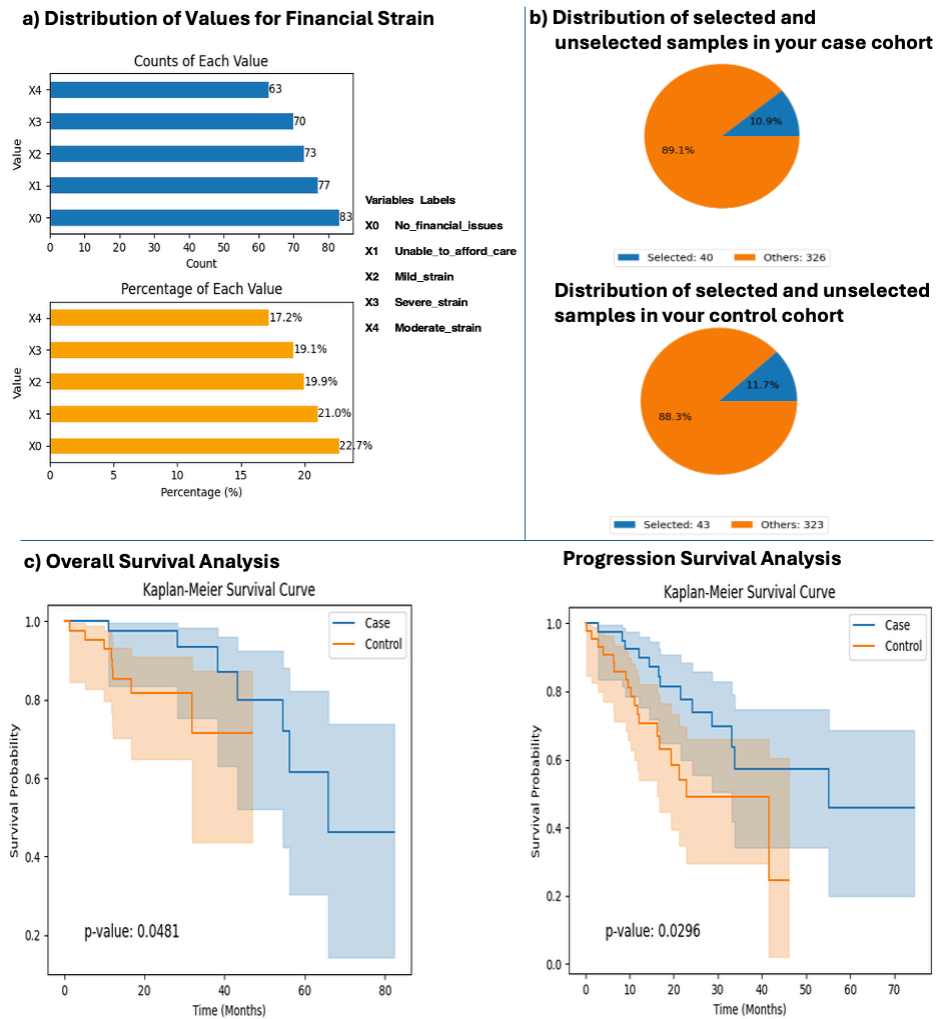
AI-HOPE-PM (Artificial Intelligence Agent for High-Optimization and Precision Medicine in Population Metrics) analysis of folinic acid, fluorouracil, and oxaliplatin–treated patients with colorectal cancer with *TP53* mutations and varying levels of financial strain.

In a prominent use case, AI-HOPE-PM analyzed data from the TCGA COAD dataset to investigate how financial strain affects outcomes among folinic acid, fluorouracil, and oxaliplatin (FOLFOX)–treated patients with CRC with *TP53* mutations (Figure 2). The analysis began by selecting the COAD dataset enriched with SDOH data, allowing users to explore attribute distributions such as financial strain. A bar chart visualization was generated, showing both the count and percentage distribution of financial strain levels across the dataset (Figure 2A). Based on user-defined filtering criteria—patients treated with FOLFOX and harboring *TP53* mutations—AI-HOPE-PM created two cohorts: a case cohort of 40 (10.9%) patients reporting mild or no financial issues and a control cohort of 43 (11.7%) patients experiencing moderate to severe financial strain, including those unable to afford care. Pie charts illustrated the proportional distribution of these cohorts within the total 366-sample dataset (Figure 2B). Once cohorts were defined, the user selected a survival analysis module. AI-HOPE-PM performed a Kaplan-Meier analysis to assess both overall and progression-free survival. The resulting survival plots demonstrated significantly shorter survival in the control group compared to the case group, with *P* values of .05 (overall survival) and .03 (progression-free survival), supported by CIs indicating statistical robustness (Figure 2C). These findings underscore AI-HOPE-PM’s ability to integrate clinical, genomic, and SDOH data through natural language–guided workflows, enabling rapid identification of clinically meaningful disparities in treatment outcomes and survival. This functionality is further supported by the multimedia demonstration with a similar query [27].

**Figure 3.**
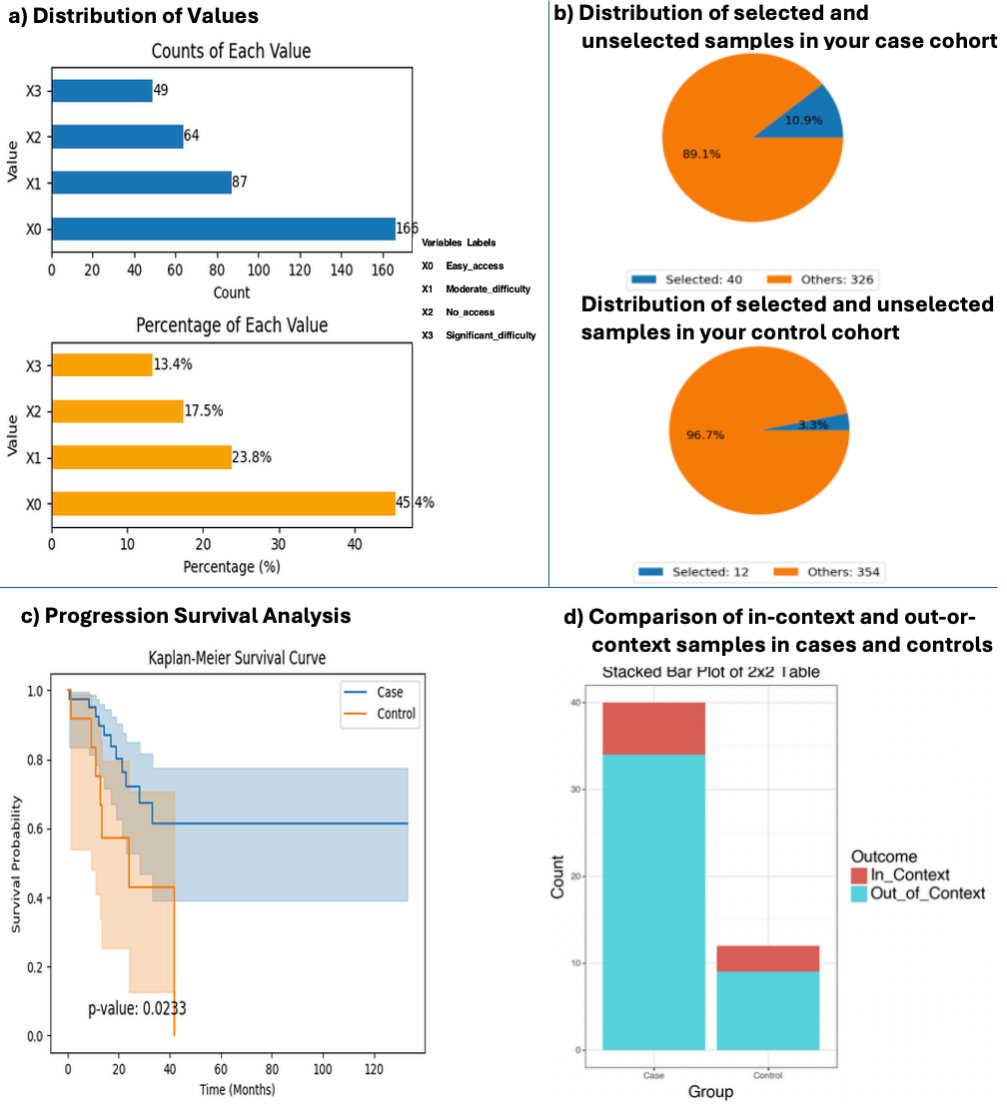
AI-HOPE-PM (Artificial Intelligence Agent for High-Optimization and Precision Medicine in Population Metrics) analysis of patients with colorectal cancer with and without *APC* mutations that have easy access to health care and treated with folinic acid, fluorouracil, and oxaliplatin.

Another case study explored the impact of *APC* mutation status among patients with CRC treated with FOLFOX and reporting easy access to health care (Figure 3). The analysis began by selecting the COAD dataset enriched with SDOH, which enabled exploration of the distribution of health care access variables. A bar chart was generated to visualize both the count and percentage of patients stratified by their reported level of health care access (Figure 3A). AI-HOPE-PM then applied user-defined filters to create case and control cohorts. The case cohort consisted of 40 (10.9%) patients who had *APC* mutations, reported easy access to health care, and received FOLFOX treatment. The control cohort comprised 12 (3.3%) patients who met the same filtering criteria except they were *APC* wild-type. Pie charts illustrated the proportional distribution of these cohorts out of the total 366 patients in the dataset (Figure 3B). After defining the cohorts, AI-HOPE-PM enabled the user to run a Kaplan-Meier survival analysis, which revealed that patients in the control group (*APC* wild-type) experienced significantly poorer progression-free survival, with a *P* value of .02, as shown in the survival plot (Figure 3C). This suggests a potential prognostic role of *APC* mutation status under standardized treatment and access conditions. Additionally, the system performed an odds ratio analysis to assess differences in ethnic representation between the cohorts. In this context, Hispanic/Latino identity was used as the comparative variable. The case cohort included 6 (15%) in-context Hispanic/Latino patients and 34 out-of-context patients, while the control cohort included 3 (15%) in-context patients and 9 out-of-context patients. The resulting odds ratio was 0.529 (95% CI 0.11-2.541), indicating a lower—but not statistically significant—representation of Hispanic/Latino individuals in the control group (Figure 3D). Together, these results reinforce AI-HOPE-PM’s ability to integrate genomic, clinical, and SDOH variables and to highlight the importance of considering ancestral background and access to care when evaluating mutation-driven outcomes in precision oncology. As shown through the multimedia demonstration with a similar query [28], the platform effectively processes complex, user-defined inputs.

In a third application, AI-HOPE-PM examined patients with early-onset CRC (age <50 y) to evaluate the impact of social support on survival outcomes among those treated with FOLFOX chemotherapy (Multimedia Appendix 1). The user began by selecting the COAD dataset containing enriched SDOH data. This enabled visualization of patient-level attributes such as age, treatment type, mutation status, and social support level. Histograms and bar plots provided an overview of the distribution of these variables across the cohort (A). The case cohort was defined using user-set criteria: patients younger than 50 years, treated with FOLFOX, and classified as having strong or moderate social support, resulting in 17 patients. This subset was visualized using pie charts to reflect the proportion of selected versus total samples (B). In parallel, the control cohort was defined with the same criteria except for social support, selecting 14 patients who reported limited or no support. A similar pie chart depicted the sample distribution for the control group (C). A Kaplan-Meier survival analysis was then conducted to assess overall and progression-free survival differences between the two groups. The survival curves revealed a statistically significant difference in progression-free survival (*P*=.02), with the control group experiencing poorer outcomes. Although the difference in overall survival did not reach statistical significance (*P*=.07), a trend toward worse survival in the control group was observed (D). To further characterize these groups, an odds ratio analysis was performed using *TP53* mutation status as the comparative context. The case group had a lower—but not statistically significant—prevalence of *TP53* mutations, resulting in an odds ratio of 0.706 (95% CI 0.208-2.396; E). These findings suggest that lower levels of social support may be linked to poorer progression-free survival and potentially associated with mutational profiles, reinforcing the importance of incorporating psychosocial variables in precision oncology—particularly in younger patients with CRC. The capabilities of AI-HOPE-PM are further demonstrated in the multimedia example of a comparable query [29].

In another analysis focused on food insecurity, AI-HOPE-PM investigated survival disparities and treatment access among patients with CRC with *APC* mutations (Multimedia Appendix 2). The analysis began with the selection of the COAD dataset integrated with SDOH, allowing the user to visualize variables such as food insecurity, treatment type, and *APC* mutation status. Histograms and bar plots summarized the distribution of these attributes across the cohort—highlighting proportions of food-insecure patients and chemotherapy exposure stratified by mutation status (A). The case cohort was defined as patients reporting no food insecurity and having an *APC* mutation, yielding 245 samples. A pie chart illustrated the representation of this subset within the overall dataset (B). The control cohort was established using the same criteria—*APC* mutation present—but selecting patients with moderate-to-severe food insecurity, resulting in 206 samples. A corresponding pie chart depicted this cohort’s proportional distribution (Multimedia Appendix 2C). A Kaplan-Meier progression-free survival analysis was performed, stratifying patients by treatment type, specifically whether they received chemotherapy. Although exact *P* values were not displayed in the figure, the survival curves showed a clear separation, suggesting poorer outcomes in food-insecure patients not receiving chemotherapy (D). These visual trends support the finding that food-insecure patients experienced significantly worse progression-free survival, as confirmed by a *P* value of .02 from the associated analysis. To further explore this disparity, an odds ratio analysis was conducted using TREATMENT_TYPE (chemotherapy vs nonchemotherapy) as the comparative context. The results revealed that food-insecure patients were less likely to receive chemotherapy, with an odds ratio of 0.356 (95% CI 0.136-1.186), indicating a potential treatment access gap (E). This analysis underscores the ability of AI-HOPE-PM to uncover how socioeconomic burden, in conjunction with genomic context, may modulate both treatment delivery and clinical outcomes in CRC. As illustrated through the multimedia demonstration of a similar query [30], the platform effectively interprets complex natural language inputs.

Sex-based disparities were explored in a separate analysis focusing on patients with CRC with limited health literacy who were treated with FOLFOX chemotherapy (Multimedia Appendix 3). AI-HOPE-PM utilized the COAD dataset enriched with SDOH and genomic annotations to assess the intersection of insurance status, tumor stage, and *KRAS* mutation status. Bar charts provided an overview of insurance coverage within the dataset, illustrating both the absolute counts and proportional distribution across different insurance categories (A). To define the cohorts, the case group was filtered to include insured patients who had *KRAS* mutations, were diagnosed at stage I or II, and received leucovorin-based chemotherapy, yielding 31 samples. This subset was visualized using a pie chart to indicate its proportion out of the total 373 samples (B). The control cohort applied identical clinical and molecular filters but included only uninsured patients, resulting in 30 samples (C). This side-by-side comparison emphasizes how insurance coverage may influence patient stratification and treatment access, even under otherwise uniform clinical conditions. An odds ratio test was performed using *KRAS* mutation status as the defining context to examine mutation prevalence differences between insured and uninsured groups. A stacked bar chart visualized the distribution of in-context (*KRAS*-mutated) versus out-of-context samples in each group. The analysis revealed a modest difference in *KRAS* mutation representation, suggesting that financial access to care could intersect with genomic profiles in ways that warrant deeper investigation (D). In a related sex-disparity analysis among patients with limited health literacy, AI-HOPE-PM defined a case cohort of 33 females and a control cohort of 41 males, both treated with FOLFOX. Odds ratio testing using *KRAS* mutation status showed that 30.3% of females and 56.1% of males were *KRAS*-mutated, yielding an odds ratio of 0.503 (95% CI 0.192-1.319; *P*=.24). Although not statistically significant, these findings suggest potential sex-based differences in *KRAS* mutation prevalence under constrained health literacy conditions and highlight the utility of AI-HOPE-PM for uncovering multidimensional disparities in cancer genomics and treatment. This process is illustrated through the multimedia demonstration of a similar query [31].

AI-HOPE-PM also facilitated analyses of nongenomic SDOH influences on CRC outcomes. In one study, the platform was used to explore how insurance status, treatment exposure, and clinical care setting affected survival among patients in the COAD dataset (Multimedia Appendix 4). The analysis began with the selection of a dataset enriched with SDOH attributes. Bar charts provided a comprehensive overview of insurance type distribution, showing both the absolute number of patients per insurance category and their relative proportions, offering insight into the socioeconomic landscape of the cohort (A). Using this context, AI-HOPE-PM defined a case cohort of 41 insured patients with the following characteristics: stage IV CRC, Hispanic/Latino ethnicity, FOLFOX treatment, and care received at a community oncology practice. A pie chart visualized the size of this cohort relative to the dataset (B). A control cohort was generated using the same clinical and demographic criteria but restricted to uninsured patients, resulting in 22 samples. The corresponding pie chart highlighted the discrepancy in sample size and access between the insured and uninsured groups (C). Following cohort definition, AI-HOPE-PM performed a Kaplan-Meier survival analysis to evaluate overall survival outcomes. The survival plots illustrated a clear separation between the two groups, with uninsured patients showing poorer survival outcomes, despite receiving similar treatments and having similar disease profiles (D). While the figure does not specify *P* values or CIs, the divergence in survival curves strongly suggests a detrimental impact of lack of insurance on patient outcomes. These findings underscore the critical role of insurance coverage in modulating survival, even when controlling for genomic, treatment, and staging variables. As demonstrated in the multimedia example using a comparable query [26], the platform accurately handles complex, user-driven inputs.

This study complements other AI-HOPE-PM findings by leveraging its capacity to integrate SDOH with clinical and genomic data to uncover disparities in CRC care and outcomes. In one analysis, the system examined the relationship between moderate to severe financial strain and CRC screening adherence, revealing that patients experiencing economic hardship were significantly less likely to participate in screening programs, highlighting a critical barrier to early detection (Multimedia Appendix 5). The analysis began with the selection of the SocialFactors_COAD dataset, enabling structured visualization of variables such as *APC* mutation status and health care access levels. Bar plots showed both the frequency of *APC* mutations and the distribution of health care access categories within the full cohort (A). A case cohort of 326 patients was created using filters for limited health care access; treatment with agents in fluorouracil, leucovorin, and oxaliplatin; and presence of *APC* mutations (mutation_status=1). A pie chart depicted their proportion relative to the total dataset (B). A control cohort of 354 patients was defined using the same criteria except for *APC* wild-type status (mutation_status=0). Their distribution was similarly visualized (C). A Kaplan-Meier progression-free survival analysis was then performed, stratified by chemotherapy treatment status and highlighting differences particularly among Hispanic/Latino patients. The survival curves revealed a noticeable separation between groups, suggesting a potential survival disadvantage linked to disparities in health care access and genomic background (D). Additionally, an odds ratio analysis evaluated treatment disparities based on chemotherapy exposure across the defined cohorts. A bar plot illustrated differences in chemotherapy receipt, reinforcing how limited access to care and mutation status may jointly influence treatment pathways and clinical outcomes (E). Other AI-HOPE-PM analyses supported these observations. One study found that patients reporting low social support or isolation had higher rates of treatment discontinuation and worse survival outcomes, consistent with psychosocial oncology literature [24]. The platform also uncovered racial and ethnic disparities in progression-free survival, with non-Hispanic White patients demonstrating better outcomes than Black and Hispanic patients, even after adjusting for treatment type and disease stage. Collectively, these results underscore the value of incorporating SDOH variables into precision medicine frameworks, enabling AI-HOPE-PM to reveal systemic inequities that might otherwise be overlooked in genomic-only analyses. The multimedia demonstration of a similar query [29] highlights the platform’s ability to interpret and execute complex, user-defined instructions.

AI-HOPE-PM demonstrated high computational efficiency, executing high-dimensional case-control studies involving over 10,000 patient records in under 1 minute. In a benchmark comparison, the platform required only 28.02 seconds to open the application, select a database, and filter a single data attribute—significantly faster than cBioPortal (58.01 s) and UCSC Xena (46.06 s). By automating the ingestion, filtering, analysis, and reporting stages, AI-HOPE-PM substantially reduced manual burden and turnaround time compared to conventional bioinformatics tools. This performance underscores its value as a scalable AI platform capable of delivering real-time, integrative data analysis to support precision oncology and health equity research.

In a comparative timing analysis, AI-HOPE-PM significantly outperformed established platforms such as cBioPortal and UCSC Xena in executing basic data query tasks. The standardized task—which included launching the application, selecting a dataset, and applying a filter based on a single data attribute—was completed in just 28.02 seconds using AI-HOPE-PM. In contrast, the same task required 58.01 seconds on cBioPortal and 46.06 seconds on UCSC Xena. These results underscore the efficiency advantages of AI-HOPE-PM’s natural language–driven, automated workflow, which streamlines multistep analyses and reduces manual input time compared to traditional GUI-based platforms.

## Discussion

### Principal Findings

This study presents the development and application of AI-HOPE-PM, a conversational AI system designed to integrate clinical, genomic, and SDOH data for precision oncology research. AI-HOPE-PM addresses key limitations in existing bioinformatics tools by enabling users to pose natural language queries that are automatically translated into executable workflows. This allows for case-control stratification and hypothesis testing that include both molecular and nonmolecular variables.

In multiple CRC case studies, AI-HOPE-PM demonstrated the ability to reveal associations between genomic alterations (eg, *TP53* and *APC* mutations), treatment exposures (eg, FOLFOX chemotherapy), and SDOH variables such as financial strain, food insecurity, health care access, and social support. These findings underscore the importance of contextualizing genomic data within broader socioeconomic and behavioral frameworks to better understand cancer disparities and inform population-relevant strategies.

### Comparison to Prior Work

Traditional tools such as cBioPortal and UCSC Xena have facilitated broad access to public cancer genomic datasets, yet they require manual, multistep filtering and operate within fixed analytical frameworks. These platforms typically lack support for SDOH integration and require a certain level of technical expertise, limiting their accessibility for noncomputational researchers and clinicians. More recent tools like CellAgent [17] and AutoBA [18] have begun to explore the use of LLMs in biomedical contexts, but their scope is generally limited to genomic analysis and does not extend to the integration of clinical or social variables essential for advancing health equity.

Our group’s prior work introduced AI-HOPE, a closed-system, LLM-driven conversational agent designed to enable integrative clinical and genomic data analyses through natural language interactions [23]. AI-HOPE allows users to perform association studies, prevalence assessments, and survival analyses on locally stored datasets while maintaining data security and interpretability. It demonstrated its capabilities by identifying well-documented associations in TCGA CRC datasets, such as the enrichment of *TP53* mutations in late-stage CRC and the association of *KRAS* mutations with poor progression-free survival in FOLFOX-treated patients. While AI-HOPE addressed the integration of clinical and genomic data, it was not explicitly designed to handle population-level equity metrics or SDOH variables.

AI-HOPE-PM builds on and significantly extends this foundation by incorporating SDOH dimensions—such as financial strain, health care access, food insecurity, and health literacy—into its analytical framework. This addition allows researchers to study cancer outcomes in a more holistic context, bridging molecular findings with real-world social environments. Furthermore, AI-HOPE-PM expands the scope of natural language query handling to accommodate multimodal stratification involving genomic, clinical, and social parameters, which is essential for addressing health disparities. By doing so, it complements AI-HOPE’s functionality while introducing new capabilities that are critical for equity-focused translational research.

### Strengths and Limitations

A key strength of AI-HOPE-PM is its ability to perform integrative, user-defined analyses through natural language queries without requiring programming expertise. This significantly reduces technical barriers for clinician-scientists and public health researchers. Importantly, the platform enables the inclusion of SDOH variables—such as financial strain, health care access, and social support—that are often absent from traditional bioinformatics workflows. Its modular architecture supports rapid cohort definition, survival modeling, and odds ratio testing across large, harmonized datasets, allowing for real-time hypothesis generation and exploratory analysis.

However, several limitations should be acknowledged. First, while this study used harmonized and simulated SDOH variables to demonstrate the platform’s functionality, the availability and quality of real-world, longitudinal SDOH data remain limited in many health care systems. This may affect the generalizability of findings and the real-world applicability of the platform. Future efforts will require integration with validated, longitudinal SDOH datasets—potentially through partnerships with clinical institutions and population health data repositories. Second, AI-HOPE-PM’s current design is optimized for structured, publicly available datasets such as TCGA, cBioPortal, and AACR GENIE. As such, its adaptability to unstructured clinical data or eHealth records is limited. While this design choice enhances reproducibility and alignment with standardized biomedical ontologies, future work should explore interoperability with clinical informatics platforms and natural language extraction from eHealth records to expand usability in health care settings. Third, this study focused exclusively on CRC datasets. As a result, findings and workflows may not be immediately generalizable to other cancer types without retraining or additional customization of the AI system. Although the architecture is designed to be adaptable, validation on other tumor types and disease areas will be essential for broader adoption. Fourth, while benchmarking analyses demonstrated strong performance compared to tools like cBioPortal and UCSC Xena, formal usability testing and prospective validation in real-world clinical and research environments were not conducted. These are planned as part of future development phases and will be critical for refining the user interface, evaluating human-AI collaboration, and assessing clinical impact. By acknowledging and addressing these limitations, future iterations of AI-HOPE-PM can be improved to better support equitable, scalable, and clinically relevant precision medicine research.

A notable limitation of the current study is the use of simulated SDOH variables rather than real-world data. While these simulated features were generated to reflect established patterns from peer-reviewed literature and public health datasets, they cannot fully replicate the variability, context-dependence, or missingness typical of empirical SDOH data collected in clinical or community settings. This limitation may impact the external validity of some findings and restrict generalizability. To address this, we are actively pursuing collaborations with health systems and community-based data partners to incorporate validated, longitudinal SDOH datasets into future deployments of AI-HOPE-PM. This planned integration will enable more accurate assessment of equity-relevant outcomes and enhance the platform’s application in real-world clinical research.

While AI-HOPE-PM achieved a high query interpretation accuracy of 92.5% during internal evaluation, several error modes were identified that merit consideration. The most frequent issues involved ambiguity in natural language input—particularly when users provided imprecise criteria for cohort selection or omitted critical parameters. Additionally, complex nested queries and nonstandard phrasing occasionally led to misinterpretation or partial execution. In a minority of cases, errors stemmed from misalignment between user terminology and the platform’s internal ontology, particularly for less common clinical or SDOH variables. To address these challenges, AI-HOPE-PM integrates clarification prompts that guide users toward more precise query formulation and supports synonym recognition for common variable names. Ongoing improvements include refining the language model’s domain specificity and expanding the internal ontology to better accommodate diverse user inputs. These enhancements are essential for improving reproducibility and user experience in real-world settings.

A key limitation of this study is the use of simulated SDOH variables rather than real-world data. While simulation allowed us to prototype and evaluate the functionality of AI-HOPE-PM under controlled conditions, it does not fully capture the complexity, heterogeneity, or potential missingness often present in real clinical and social datasets. To address this limitation, we have developed and released an open-source Python script [25] that transparently outlines our simulation methodology. Additionally, we are actively working on the integration of real-world SDOH data through ongoing projects [32], which is sequencing and characterizing tumors from 500 Hispanic/Latino patients in the Los Angeles catchment area. These datasets will allow us to test AI-HOPE-PM’s performance in real clinical environments and refine its capacity to analyze authentic, population-specific SDOH variables in future iterations.

To address this limitation, we acknowledge that the current evaluation of AI-HOPE-PM using 100 natural language queries—while carefully curated by physician-scientists, public health researchers, biostatisticians, and bioinformaticians to reflect real-world clinical and translational scenarios—represents an early validation phase. These queries were intentionally designed to ensure clinical accuracy, relevance, and internal consistency. However, we recognize the importance of expanding evaluation to include a broader and more diverse group of end users across different levels of expertise. Future iterations of AI-HOPE-PM will incorporate structured feedback from clinicians, public health researchers, and community health stakeholders. This participatory approach will help identify diverse interaction patterns, reduce potential biases, and enhance the platform’s interpretive capacity over time.

### Future Directions

Future development of AI-HOPE-PM will focus on several enhancements. First, expanding support for additional omics layers [32], including spatial biology [33] and single-cell [34-36], could improve the platform’s applicability to emerging areas in systems oncology. Integration with federated learning frameworks may also enable secure, institution-specific model updates without compromising patient privacy. Moreover, enhancing the system’s ability to handle longitudinal data, including treatment timelines and SDOH trajectories, will be critical for supporting causal inference and policy-relevant research [37-42].

Future iterations of AI-HOPE-PM will prioritize the integration of more inclusive and representative genomic datasets to enhance the platform’s utility across diverse patient populations. While the current analyses rely on publicly available sources such as TCGA and cBioPortal—which are known to underrepresent racial and ethnic minorities—there have been encouraging advances in improving dataset diversity, particularly in CRC cohorts submitted by major US cancer centers. Notably, several ongoing initiatives aim to sequence and characterize tumors from historically underrepresented populations, including Hispanic/Latino patients with CRC [32]. These datasets, once publicly released, will be incorporated into AI-HOPE-PM to improve its generalizability and relevance in addressing cancer health disparities. This aligns with our overarching mission to develop equity-focused precision oncology tools that are responsive to the needs of all communities.

In this study, benchmarking primarily focused on task completion time—measuring the duration to execute standard bioinformatics queries across AI-HOPE-PM, cBioPortal, and UCSC Xena. While AI-HOPE-PM demonstrated superior efficiency due to its natural language automation, we acknowledge that this assessment does not encompass analytical output comparison. Future benchmarking studies will evaluate not only speed but also reproducibility and concordance of statistical results, including survival curves, odds ratios, and subgroup analyses. This expanded evaluation will ensure that AI-HOPE-PM delivers results comparable in accuracy and robustness to established platforms, further supporting its utility for translational cancer research.

A preliminary usability assessment was conducted during an internal pilot deployment involving five clinician-scientists and three public health researchers. Participants were asked to complete common clinical-genomic queries using AI-HOPE-PM and provide structured feedback on system usability, interpretability of outputs, and ease of query formulation. Feedback indicated that users found the natural language interface intuitive and appreciated the automation of statistical analyses without coding. Suggestions for improvement included refining terminology prompts and expanding visualization customization. These insights have been incorporated into the current version of AI-HOPE-PM, and a formal usability study with a larger and more diverse cohort is currently underway to systematically evaluate accessibility, performance, and user satisfaction.

To enhance accessibility and promote broader adoption, particularly in resource-constrained environments, we are actively exploring deployment strategies that reduce local infrastructure requirements. Although the current AI-HOPE-PM system benefits from graphics processing unit acceleration for large-scale genomic analyses, the core functionalities—including query interpretation, basic statistical modeling, and report generation—can be executed on standard central processing unit-based systems. Additionally, we are developing a lightweight web-hosted version of the platform with backend support on scalable cloud infrastructure, enabling institutions with limited computational resources to access AI-HOPE-PM through a browser without the need for specialized hardware. Future iterations will also offer modular processing options that allow users to select compute-intensive features based on available resources.

User-centered evaluations—including usability studies with diverse researchers and clinicians—are planned to better understand the platform’s accessibility and impact in real-world settings. Additionally, collaborations with community-based research initiatives may help validate AI-HOPE-PM’s role in addressing health disparities and improving equity in precision medicine.

AI-HOPE-PM was developed with scalability and accessibility in mind, including potential deployment in resource-constrained settings. The system can be installed and executed locally, eliminating the need for high-bandwidth internet or continuous cloud access. While graphics processing unit acceleration can enhance performance for large-scale queries, the platform’s modular backend supports central processing units–only configurations for smaller datasets and standard analyses. Ongoing optimization efforts aim to further reduce computational overhead through lightweight LLM variants and model compression techniques. These features support broader adoption across diverse institutional environments, including low-resource clinical and research settings.

A key consideration for the broader adoption of AI-HOPE-PM is the potential for language bias and variability in natural language queries. While the current version of the platform is optimized for English-language input, this may limit accessibility for nonnative English speakers or introduce semantic variability that could affect interpretation. To mitigate this, AI-HOPE-PM employs a domain-specific ontology with synonym recognition and structured clarification prompts that guide users toward standardized, interpretable input. These features reduce the likelihood of misinterpretation and increase the reliability of query processing. Nonetheless, we recognize the importance of supporting diverse linguistic backgrounds in biomedical research. Future iterations of the platform will integrate multilingual capabilities and undergo structured usability evaluations in non–English-speaking populations to ensure equitable utility and minimize language-related inequities in research engagement.

## Supplementary material

10.2196/76553 Multimedia Appendix 1 AI-HOPE-PM (Artificial Intelligence Agent for High-Optimization and Precision Medicine in Population Metrics) analysis of patients with early-onset colorectal cancer treated with folinic acid, fluorouracil, and oxaliplatin and varying levels of social support.

10.2196/76553 Multimedia Appendix 2 AI-HOPE-PM (Artificial Intelligence Agent for High-Optimization and Precision Medicine in Population Metrics) analysis of patients with colorectal cancer with and without chemotherapy treatment, food security, and APC mutations.

10.2196/76553 Multimedia Appendix 3 AI-HOPE-PM (Artificial Intelligence Agent for High-Optimization and Precision Medicine in Population Metrics) analysis of patients with colorectal cancer with KRAS mutations in the context of insurance coverage and tumor stage.

10.2196/76553 Multimedia Appendix 4 AI-HOPE-PM (Artificial Intelligence Agent for High-Optimization and Precision Medicine in Population Metrics) analysis of survival outcomes in patients with colorectal cancer with different insurance and treatment profiles.

10.2196/76553 Multimedia Appendix 5 AI-HOPE-PM (Artificial Intelligence Agent for High-Optimization and Precision Medicine in Population Metrics) stratification of patients with colorectal cancer by health care access, *APC* mutation, and ethnicity for survival and treatment disparity analysis.

## Abbreviations

AI: artificial intelligence
AI-HOPE-PM: Artificial Intelligence Agent for High-Optimization and Precision Medicine in Population Metrics
CRC: colorectal cancer
FOLFOX: folinic acid, fluorouracil, and oxaliplatin
GUI: graphical user interface
LLM: large language model
SDOH: social determinants of health
TCGA: The Cancer Genome Atlas
