# Supplementary material for: Conversational Artificial Intelligence for Integrating Social Determinants, Genomics, and Clinical Data in Precision Medicine: Development and Implementation Study of the AI-HOPE-PM System
Source: JMIR Bioinform Biotechnol. 2025 Oct 10;6:e76553. doi: 10.2196/76553 (PMC12513684; doi:10.2196/76553)
Supplement: Multimedia Appendix 4 [file bioinform-v6-e76553-s004.docx]

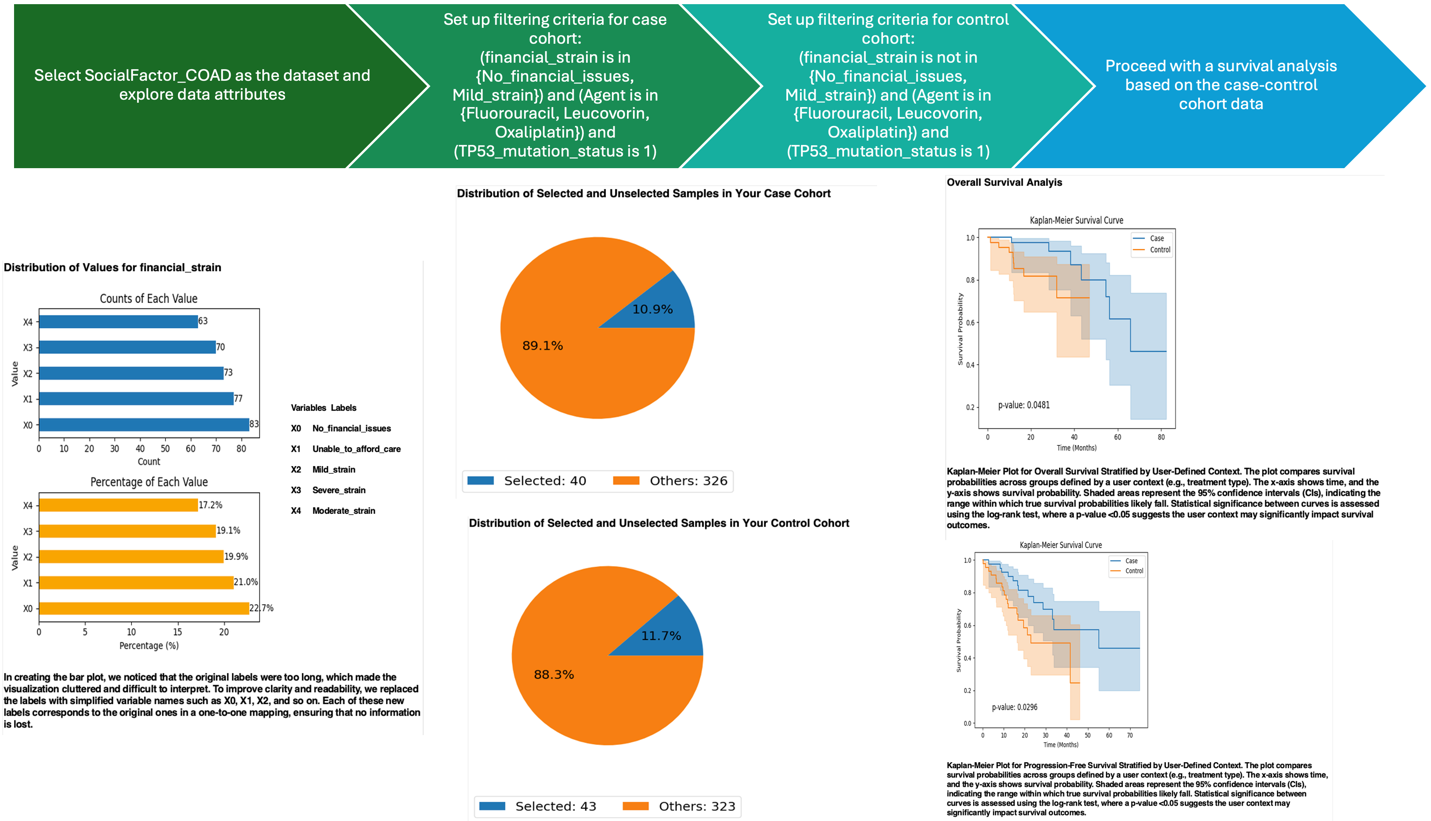


d)

c)

b)

a)

**Multimedia Appendix 4. AI-HOPE-PM Analysis of Survival Outcomes in CRC Patients with Different Insurance and Treatment Profiles.**
